# Pharmaceutical Pollution Alters the Structure of Freshwater Communities and Hinders Their Recovery from a Fish Predator

**DOI:** 10.1021/acs.est.4c02807

**Published:** 2024-07-25

**Authors:** Marcus Michelangeli, Jake M. Martin, Stephanie Robson, Daniel Cerveny, Robert Walsh, Erinn K. Richmond, Michael R. Grace, Jack A. Brand, Michael G. Bertram, Susie S. Y. Ho, Tomas Brodin, Bob B. M. Wong

**Affiliations:** † School of Environment and Science, Griffith University, Nathan 4111, Australia; ‡ Department of Wildlife, Fish and Environmental Studies, Swedish University of Agricultural Sciences, Umeå 901 83, Sweden; § School of Biological Sciences, Monash University, Melbourne 3800, Australia; ∥ Department of Zoology, Stockholm University, Stockholm 114 18, Sweden; ⊥ Water Studies Centre, School of Chemistry, Monash University, Melbourne 3800, Australia; # University of South Bohemia in Ceske Budejovice, Faculty of Fisheries and Protection of Waters, South Bohemian Research Center of Aquaculture and Biodiversity of Hydrocenoses, Zatisi 728/II, Vodnany 389 25, Czech Republic; ¶ Australian Waterlife, 55 Vaughan Chase, Wyndham Vale, Victoria 3024, Australia; ∇ Environmental Protection Authority Victoria, EPA Science, Macleod, Victoria 3085, Australia; ○ Institute of Zoology, Zoological Society of London, London NW1 4RY, U.K.

**Keywords:** primary productivity, chemical contaminants, fluoxetine, invasive species, pharmaceuticals

## Abstract

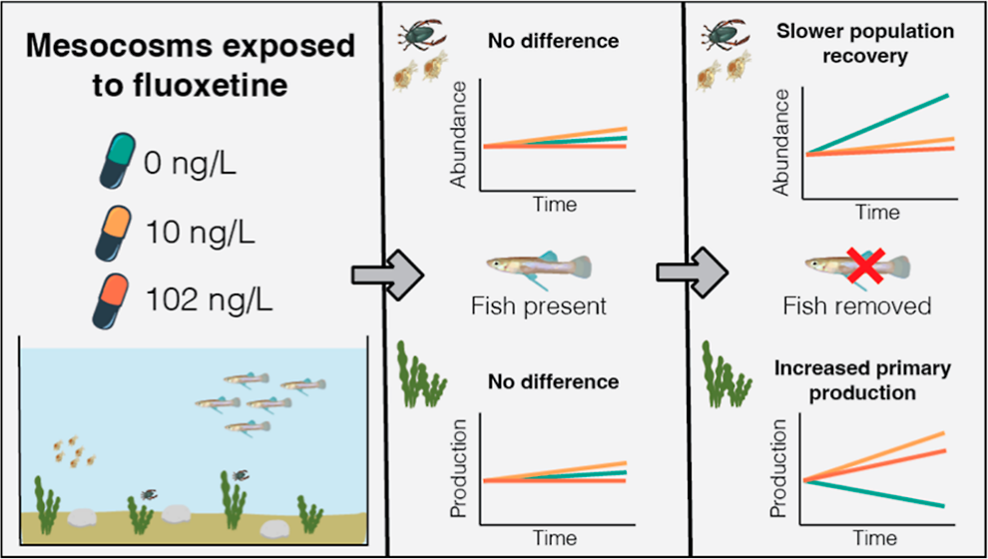

Freshwater ecosystems are under threat from rising pharmaceutical pollution. While such pollutants are known to elicit biological effects on organisms, we have limited knowledge on how these effects might cascade through food-webs, disrupt ecological processes, and shape freshwater communities. In this study, we used a mesocosm experiment to explore how the community impacts of a top-order predator, the eastern mosquitofish (*Gambusia holbrooki*), are mediated by exposure to environmentally relevant low (measured concentration: ∼10 ng/L) and high concentrations (∼110 ng/L) of the pervasive pharmaceutical pollutant fluoxetine. We found no evidence that exposure to fluoxetine altered the consumptive effects of mosquitofish on zooplankton. However, once mosquitofish were removed from the mesocosms, zooplankton abundance recovered to a greater extent in control mesocosms compared to both low and high fluoxetine-exposed mesocosms. By the end of the experiment, this resulted in fundamental differences in community structure between the control and fluoxetine-treated mesocosms. Specifically, the control mesocosms were characterized by higher zooplankton abundances and lower algal biomass, whereas mesocosms exposed to either low or high concentrations of fluoxetine had lower zooplankton abundances and higher algal biomass. Our results suggest that fluoxetine, even at very low concentrations, can alter aquatic communities and hinder their recovery from disturbances.

## Abstract 2

Little is known about the effects of pharmaceutical pollution on freshwater communities. Our study finds that the common antidepressant fluoxetine can alter their structure and hinder community recovery from environmental disturbances.

## 1 Introduction

The health of freshwater ecosystems around the globe is under threat from a range of human-induced disturbances.^1,2^ One such threat is pharmaceutical pollution, with over 900 different pharmaceuticals having now been detected in aquatic environments worldwide, including psychotropics, antibiotics, painkillers, antihistamines, and anti-inflammatory drugs.^3−5^ Many pharmaceuticals and their byproducts are continuously discharged into waterways from domestic, industrial, and agricultural sources.^6^ Moreover, as the world’s population grows and cities expand, pharmaceutical pollution is expected to continue to rise substantially into the future.^7^ An urgent research priority is therefore to identify the hazards that pharmaceutical contaminants may pose to the structure and function of freshwater ecosystems.^1,8^

While detected concentrations of pharmaceuticals in surface and ground waters are typically low (in the ng/L range), chronic exposure to these pollutants may elicit sublethal effects on organisms that could act as powerful drivers of ecological instability.^9,10^ Several studies have highlighted that exposure to pharmaceutical contaminants may have the capacity to directly disrupt key ecosystem processes by altering community composition and functionality.^11−16^ For instance, exposure of artificial streams to the stimulant drug amphetamine changed the composition of bacterial and diatom assemblages and decreased biofilm productivity.^14^ Exposure to pharmaceuticals may also indirectly affect population and community dynamics via their influence on organismal behavior.^10,17−19^ Many pharmaceuticals are designed to exert physiological effects at low concentrations and target biological receptors that are conserved among species, meaning that they can elicit unintended effects on nontarget organisms.^20^ For example, exposure to psychoactive pharmaceuticals has been shown to alter the foraging behavior and feeding rate of dragonfly larvae (*Aeshna spp*.), which is a common top-order predator in freshwater food-webs.^21,22^ Changes to consumer–resource interactions, such as predation rates, can alter regulatory controls between different trophic levels, leading to cascades that have important effects on aquatic community structure and energy flow.^23,24^ However, while many studies have reported biological effects of pharmaceuticals on organisms at different trophic levels, we have limited knowledge on how these effects might cascade through food-webs and shape freshwater communities.^8^

Selective serotonin reuptake inhibitors (SSRIs) are one of the most frequently detected pharmaceutical classes in aquatic environments. SSRIs are primarily prescribed to treat depression in humans and function by inhibiting the serotonin transport molecule (or SERT), thereby prolonging serotonergic signaling.^25^ Fluoxetine (marketed as Prozac) is one of the world’s most widely prescribed SSRIs. It is frequently detected in aquatic environments, with concentrations in surface waters typically ranging from less than 1 to 350 ng/L.^26^ Fluoxetine has been found to bioaccumulate in food webs and elicit effects on a range of organisms.^27,28^ For example, fluoxetine has been shown to alter the growth and production of biofilms^16,29^ and microalgae,^30^ and induce reproductive and physiological changes in both macro- and microinvertebrates.^31,32^ Furthermore, fluoxetine has been found to induce behavioral changes in wildlife, even at low concentrations (e.g., <20 ng/L^33,34^). For instance, fluoxetine has been shown to alter behaviors related to activity, predator avoidance, and foraging in fish and invertebrates.^35−38^ Such findings suggest that fluoxetine has the potential to change consumer–resource interactions, which would likely have significant consequences for other community processes.

In this study, we used a mesocosm experiment to examine how environmentally relevant low (nominal concentration: 30 ng/L) and high concentrations (nominal concentration: 300 ng/L) of fluoxetine impact the structure and dynamics of a simple aquatic community. Specifically, we measured the effects of fluoxetine on zooplankton abundance, biofilm and seston biomass and metabolism (chlorophyll *a*, gross primary production, and community respiration (CR)), and nutrient concentrations, both in the presence of a top-order predator, the eastern mosquitofish (*Gambusia holbrooki*), and after its removal. This latter aspect of our study allowed us to explore how fluoxetine influences the response of aquatic communities following release from a top-order predator. Fish often play significant consumer roles within aquatic food webs but are rarely considered in community ecotoxicology studies. The eastern mosquitofish is an extremely resilient and widespread species, occupying diverse freshwater systems that are often close to urban and rural hubs, many of which are likely exposed to elevated concentrations of pharmaceutical pollutants.^5^ The species has become invasive on multiple continents and is known for displacing native fish species and exerting top–down pressure on aquatic food webs.^39^ This is particularly true for plankton-based wetland communities, where mosquitofish often have strong consumptive effects on zooplankton and small invertebrate populations.^40−42^ Therefore, we expected that the introduction of mosquitofish into our mesocosms would exert strong consumptive effects on zooplankton, allowing us to explore how fluoxetine might mediate this top–down pressure and its consequences for lower trophic levels.

In line with recent studies suggesting that fluoxetine can affect different trophic levels and ecosystem parameters, we hypothesized that mesocosm communities exposed to fluoxetine would structurally diverge from control mesocosms both in the presence and absence of mosquitofish. Given that fluoxetine has previously been shown to alter the activity and foraging behavior of mosquitofish,^37,43−45^ we hypothesized that fluoxetine would alter mosquitofish impacts on zooplankton, leading to differences among treatments in the abundance of primary consumers and producers. However, with very little prior knowledge about how the effects of fluoxetine might cascade across trophic levels, we had no *a priori* directional predictions about how aquatic communities would ultimately differ between treatments.

## 2 Methodology

### 2.1 Mesocosm Setup

The experiment was conducted at the Jock Marshall Reserve, Monash University, Victoria, Australia (37°54′34.92″ S, 145°8′23.6328″ E). Twelve mesocosms (360 L, 100 cm length × 60 cm height × 60 cm width; made from food-grade stainless steel) were established in a 2 × 6 rectangular grid outdoors and filled with 270 L of water. Each mesocosm was assigned to one of three fluoxetine exposure treatments: solvent control, low fluoxetine (nominal concentration: 30 ng/L) or high fluoxetine (nominal concentration: 300 ng/L); for further details on treatment concentrations and application see Section 2.2.3.. Treatment groups were assigned so that they were distributed evenly across the grid to minimize spatial and environmental variability (e.g., light availability) among treatments. Each mesocosm had a lid constructed from wire mesh and transparent polycarbonate sheeting to allow sunlight but prevent any potential emigration and immigration of macroinvertebrates and other biota.

First, two large stock tanks (110 cm × 55 cm) were seeded with phytoplankton, a mixture of limnetic species, and leaf material (primarily composed of Eucalyptus spp.), which were collected from a nearby permanent local wetland at Monash University that was free of mosquitofish (37°54′35.6″ S 145°8′24.6″ E). Two weeks later, we seeded each mesocosm with 30 L of water and 400 g of wet leaf material from these stock tanks. We then added 32 clean, small ceramic tiles (5 × 5 cm) into each mesocosm as substrate for the colonisation of biofilm. Tiles were laid out in two 4 × 4 grids, with one grid on the left side of the mesocosm and the other on the right side. Water levels in the mesocosms were maintained at 270 L throughout the experiment.

### 2.2 Experimental Design

**Figure 1:**
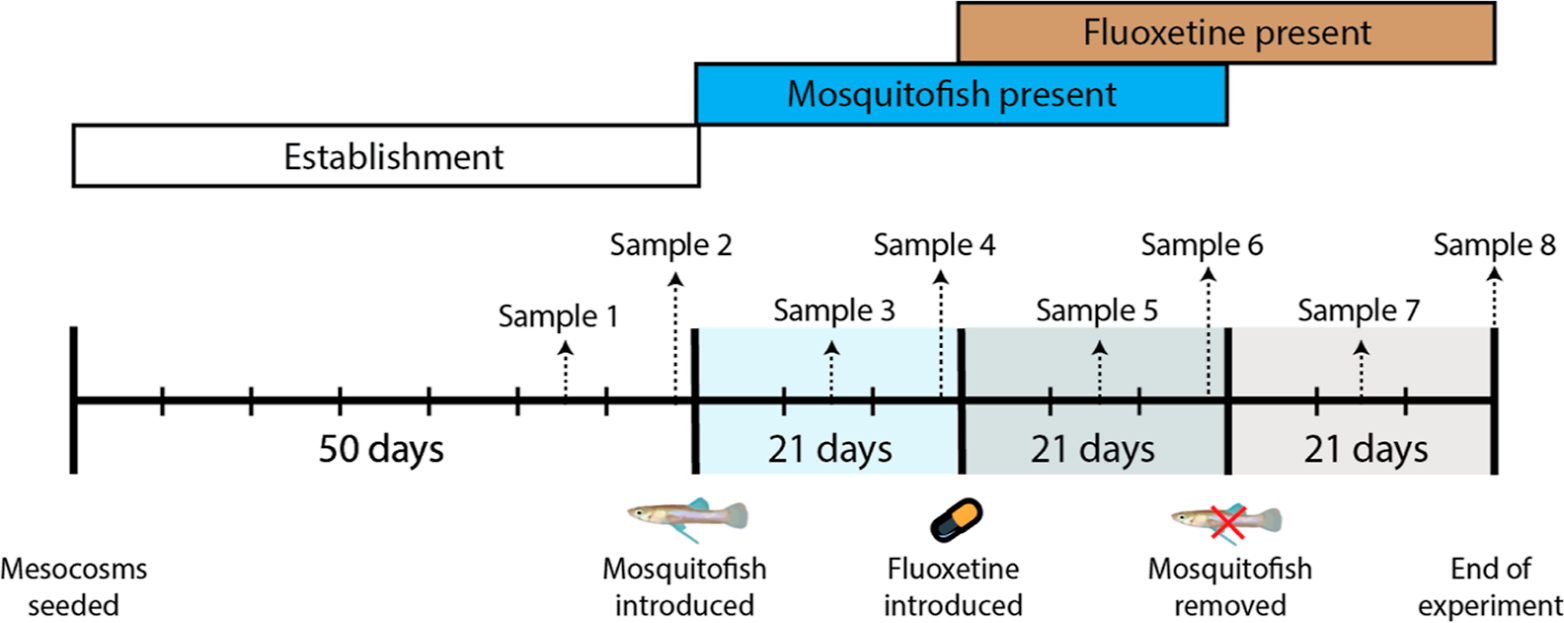
Experimental timeline over the 113 day study duration. Large vertical lines represent the beginning of key experimental stages (i.e., establishment, mosquitofish introduction, fluoxetine introduction, and post mosquitofish removal). Small vertical lines represent weeks within the experiment. Samples were taken regularly throughout the experiment to monitor for changes in zooplankton communities and primary productivity (see Section 2.3 for details).

The experiment was conducted over a period of 113 days (between 27 August and 17 December 2020) and comprised four stages: establishment (50 days), mosquitofish introduction (21 days), fluoxetine introduction (21 days), and post mosquitofish removal (21 days) (Figure 1). This sequential experimental design allowed us to (1) ensure planktonic communities were established and monitored ahead of the introduction of mosquitofish (i.e., the establishment stage), (2) examine the possible impact of mosquitofish on lower trophic levels (i.e., mosquitofish introduction), (2) determine whether and how exposure to fluoxetine mediates the impact of mosquitofish on these trophic levels (i.e., fluoxetine introduction), and (3) examine the potential influence that fluoxetine exposure has on the recovery of aquatic communities after the removal of mosquitofish (i.e., post mosquitofish removal). This experimental design and approach was adapted from a previous mesocosm study working within the same wetland system.^40^

#### 2.2.1 Establishment Stage

We allowed 50 days for mesocosms to develop stable zooplankton and algal communities prior to the introduction of mosquitofish. During this period, mesocosms were sampled twice to monitor community parameters and establish a baseline for comparing community dynamics following the introduction of mosquitofish. On October 13th 2020, due to low phytoplankton growth, we added nutrients into each mesocosm (16.5 mg K_2_HPO_4_, 213 mg of KNO_3_) to facilitate growth.

#### 2.2.2 Mosquitofish Introduction

After the mesocosms were allowed to establish for 50 days without fish (i.e., the establishment stage), we introduced a group of five adult male mosquitofish into each mesocosm. The chosen mosquitofish density was based on the results of a past survey of four seminatural ponds at Monash University that found a *Gambusia* density of 27 ± 3 (mean ± s.e.) fish m^–3^—equating to a possible density of 7 fish per 270 L (i.e., the water volume of our mesocosms).^40^ Mosquitofish were collected from the Science Centre Lake (37°54′28.692″ S, 145°8′16.872″ E) at Monash University and were housed in laboratory conditions for 45 days prior to their introduction into the mesocosms. During this housing period, mosquitofish were acclimated with their group (i.e., five fish) in glass holding tanks (60 cm length × 30 cm height × 30 cm width, 24–26 °C, 12:12 h light/dark regime), and were fed *ad libitum* once daily with either commercial pellets (Otohime Hirame larval diet 580–910 μm) or frozen *Chironomidae* larvae. Note that there was no supplemental feeding of mosquitofish once in the mesocosms. We performed 30% water changes on all housing tanks once per week. Mosquitofish groups were randomly assigned to a mesocosm. For each treatment group, we also maintained a subset of mosquitofish in the laboratory that were used to replace any mosquitofish in the mesocosms that died during the experiments. These spare mosquitofish were exposed to the same treatment conditions as mosquitofish in the mesocosms (see Section 2.2.3). During the experiment, four mosquitofish died in the mesocosms and were immediately replaced (control: *n* = 1; low fluoxetine: *n* = 2; high fluoxetine: *n* = 1).

#### 2.2.3 Fluoxetine Introduction

Mosquitofish were present in the mesocosms for 21 days before fluoxetine was introduced. Nominal fluoxetine concentrations (30 and 300 ng/L) within the exposure mesocosms were maintained by dosing the system twice weekly until the end of the experiment (Figure 1). Fluoxetine stock solutions were prepared by dissolving fluoxetine hydrochloride (Sigma-Aldrich; CAS: 56296-78-7) in methanol (98%) at 6 and 60 μg/mL for the low and high exposure treatments, respectively. A dose comprised a 1 mL aliquot of stock solution (i.e., 6 or 60 μg) diluted in 1000 mL of reverse osmosis water. In the case of control tanks, a solvent solution was added (1 mL of methanol in 1000 mL of reverse-osmosis water). The dosing regime for this experiment was based on protocols from a similar-scale mesocosm experiment using fluoxetine.^46^ Fluoxetine concentrations were selected to mirror the levels typically found in polluted surface waters (low-fluoxetine treatment), and effluent-dominated systems (high-fluoxetine treatment).^26^

#### 2.2.4 Post Mosquitofish Removal

Following 42 days in the mesocosms, and 21 days after the introduction of fluoxetine, mosquitofish were removed from the mesocosms. Mesocosms (low and high) continued to be dosed with fluoxetine—as well as the solvent solution, for control tanks—for another 21 days, until the end of the experiment (Figure 1).

### 2.3 Sample Collection and Processing

We collected regular samples from the mesocosms to monitor changes in zooplankton communities and primary productivity throughout the experiment. Samples were collected at two time points within each stage of the experiment: 11 days after the beginning of each stage, and the last day of each stage (i.e., two measurements per stage, eight total measurements throughout the entire experiment; Figure 1).

#### 2.3.1 Zooplankton

Water samples were taken for the identification and counting of zooplankton following similar methods as outlined in.^40^ Specifically, for each sample, mesocosms were divided into six quadrants, and, using a random number generator, we randomly selected a quadrant to take a 400 mL mid water column sample. This water sample was then decanted into a 250 mL bottle and sieved using a 20 μm filter into a falcon tube. Samples were maintained in 90% ethanol. Dissecting and compound microscopes were used to count and identify all taxa to a minimum of family level (typically, genus) by an observer (R.W.) who was blind to treatment.

#### 2.3.2 Biofilm and Seston Sampling

We sampled successional biofilms by taking two tiles from each mesocosm (one from the left side, and one from the right). Tiles were selected based on a random number generation process (from 1 to 16). Tiles were broken into two approximately equal segments, and each segment was placed into a 70 mL, septum-lidded jar containing a mid water column sample from the mesocosm. We measured gross primary production (GPP) and CR using light/dark incubations. We filled gas tight-jars with mesocosm water and one tile segment from each replicate mesocosm. An additional jar was filled with only mesocosm water to isolate tile biofilm metabolism from water column metabolism. To measure tile biofilm GPP, an initial measurement of dissolved oxygen (DO) and water temperature was taken using a calibrated FireString Optical DO probe (Pyroscience). We then resealed jars ensuring to eliminate all air bubbles and placed them back in their respective mesocosms to incubate in sunlight for a period of 3 h after which the DO was remeasured. Similarly, jars were incubated for 3 h in the dark, with DO measured at the start and end in order to determine CR. Following GPP and CR analysis, the tile substrates were scrubbed to form an algal slurry. Slurries were filtered (Advantec GF-75 47 mm), then the filter paper was frozen while awaiting chlorophyll *a* (*Chl-a*) analysis. Chlorophyll *a* was extracted by immersing filters in acetone in the dark for 24 h then measured using a Hitachi U 2800 UV–visible spectrophotometer. We also measured *Chl-a*, GPP, and CR from samples taken from the mid water column (i.e., including suspended materials or seston) using the methods detailed above. Water column GPP and CR were subtracted from total GPP and CR to determine the GPP and CR of the tile substrate. Tile surface area was determined using ImageJ imaging software (National Institutes of Health and the Laboratory for Optical and Computational Instrumentation). Due to an error that occurred during the sample incubations, we were unable to provide accurate measures of CR during the establishment period.

#### 2.3.3 Nutrients and Other Water Quality Parameters

Temperature, pH, and DO were measured in the mesocosms mid water column in the morning (at approximately 11:00 am) each week using a precalibrated Horiba U-10 Water Quality Checker (see Table S1 in the Supporting Information for the summary statistics for these parameters). We also measured DO concentrations (mg O_2_/L) at sunrise and sunset each week.

Water samples were also collected throughout the experiment to measure concentrations of nitrate + nitrite (NO_*x*_), filterable reactive phosphorus (FRP), and ammonia (NH_3_). Nutrient analysis was conducted blind to treatment by the Water Studies Centre at Monash University by using flow injection analysis (APHA 1998) with detection limits of 1 μg/L of N or P. Quality control procedures (including spike recoveries and standard reference materials) were employed in every sample batch.

### 2.4 Analytical Verification of Fluoxetine Concentrations

#### 2.4.1 Water

During the fluoxetine treatment stages, water samples (40 mL) were taken weekly (24 h after dosing) from mesocosms within the low and high exposure treatments, and from half of the mesocosms in the control treatment (selected randomly), to quantify water concentrations of fluoxetine. Water samples were analyzed by Envirolab Services (MPL Laboratories; NATA accreditation: 2901; accredited for compliance with ISO/IEC: 17025). Analysis was performed using liquid chromatography-tandem mass spectrometry (LC–MS/MS, Shimadzu 8050 LCMSMS), with a minimum detection limit of 2 ng/L. For more details on this protocol, see the Supporting Information in.^34^

#### 2.4.2 Macroinvertebrates

At the conclusion of the experiment, the mesocosms were drained and the remaining macroinvertebrates were sampled for verification of fluoxetine concentrations in their tissue. Specifically, from each mesocosm, we collected individuals from the following family groups, if present: Corixidae, Physidae, Notonectidae, and Chironomidae. We chose these groups because they were present in almost all mesocosms. Invertebrate specimens were carefully rinsed with distilled water to remove any foreign material, and then dried for 24 h at 60 °C. We prepared invertebrates samples for pharmaceutical extraction for each family group by placing between 3 and 10 individuals in separate sterile microcentrifuge tubes. Composite samples were necessary to meet the minimum mass required for extraction (2 mg d.w.). The samples were then pretreated by adding 5 ng of isotopically labeled internal standard (fluoxetine-*d*_5_, CAS 1173020-43-3), as well as 1.5 mL of acetonitrile, and extracted as described previously.^47,48^ In short, tissue samples underwent repeated solvent extraction, followed by evaporation of the supernatant, and its reconstitution (methanol), resulting in 150 μL of the final sample. Samples were then kept frozen at −18 °C until LC–MS/MS analysis using a triple stage quadrupole mass spectrometer (TSQ Quantiva, Thermo Scientific, San Jose, CA) operating with a heated-electrospray ionization ion source.

### 2.5 Morphological Measurements

Mosquitofish were weighed and measured for standard length (snout to caudal peduncle) both prior to their release into the mesocosms and immediately after their removal from the mesocosms. Using these weight and length measurements, we also calculated a scaled mass index as a proxy for body condition for each mosquitofish following the guidelines outlined in.^49^ Briefly, we ran a standard major axis regression (SMA, using the R package smatr;^50^) of log weight and log standard length, and used the resulting beta coefficient with mean standard length to calculate a scaled mass index for each individual mosquitofish.

## 3 Statistical Analyses

### 3.1 Model Procedure and Fitting

We conducted all data processing and analysis in R.^51^ All models were analyzed in a Bayesian framework using the *brms* package,^52^ an interface to Stan^53^ that uses a No–U-Turn Markov chain Monte Carlo algorithm. As recommended by^54^ we used regularising priors to avoid model overfitting. Models were run with four chains, and 2000 iterations with a 1000 warm-up. All models converged with low among-chain variability (Rhat = 1), and model fit was checked using posterior predictions. Priors were evaluated via prior predictive checks. Details about prior specification, model structure, and model sample sizes can be found in the Supporting Information (Table S2).

Because we were specifically interested in how communities responded to each experimental stage, and how this response differed among the control and fluoxetine treatments, for model predictions we report posterior mean percent change (with posterior 95% credibility intervals; CIs) in community end points between experimental stages for each treatment. For some community end points, we also report the model-predicted mean pairwise difference (with posterior 95% CIs) between treatment groups. Tables showing model parameter estimates (reported as posterior means with 95% CIs) and sample sizes for each analysis can be found in the Supporting Information. We produced figures using the *ggplot2*(55) and *tidybayes*(56) packages.

### 3.2 Changes in Fish Morphology

To analyze how mosquitofish morphology and body condition changed in the mesocosms, and whether this response was dependent on the control and fluoxetine treatments, we used linear mixed-effects models (LMMs) with a Gaussian error distribution. These models included treatment (3 levels: control, low fluoxetine, high fluoxetine), experimental stage (2 levels: before introduction and postremoval from mesocosms), and their interaction. Mesocosm ID was included as a random intercept to account for repeated measures and environmental variation among mesocosms.

### 3.3 Changes in Community Enpoints

To determine how mesocosm community endpoints responded to each experimental stage, and whether this response differed among control and fluoxetine treatments (i.e., after the fluoxetine exposure was introduced), we used a series of mixed-effects models. Specifically, to analyze the response of total zooplankton abundance (count data), we used a generalized linear mixed-effects model with a Negative-Binomial error distribution. To analyze the treatment response of biofilm and seston *Chl-a*, GPP, CR, and nutrients (ammonia and FRP) we used LMMs with a Gaussian error distribution. CR was square-root transformed, and FRP and ammonia were log_10_-transformed, prior to modeling. Quantifiable concentrations of NO_*x*_ were only detected during the establishment stage (see Section 4.3.4below) and thus we did not consider this end point in our statistical analysis.

Predictor variables for these models included treatment (3 levels: control, low fluoxetine, high fluoxetine), experimental stage (four levels: establishment, mosquitofish introduction, fluoxetine introduction, post mosquitofish removal), a treatment-by-stage interaction, sampling period (2 observations per experimental stage), and the average body size of the mosquitofish group within a given mesocosm. This latter predictor was standardized (by subtracting the mean and dividing by the standard deviation) to assist in model interpretation and was included in models to account for variation in mosquitofish size among the mesocosms. Mesocosm ID was also included as a random intercept in these models.

## 4 Results

### 4.1 Analytical Verification of Fluoxetine Concentrations

#### 4.1.1 Water Column

The mean measured exposure concentrations in water for the low- and high-fluoxetine treatments were 9.62 ng/L (standard deviation = 7.71, concentration range: 5.2–39, *n* = 20) and 102 ng/L (standard deviation = 71.4, concentration range: 33–310, *n* = 20), respectively (Table S3). There was no evidence of fluoxetine contamination in the control mesocosms, although we did detect a low level of fluoxetine (3.6 ng/L) in the final water sample (i.e., a sample taken on the last day of the experiment) from a single control mesocosm. A backup final water sample from this mesocosm detected no fluoxetine contamination. We also detected no fluoxetine contamination in the tissue of macroinvertebrates from the control mesocosms. Taken together, this suggests that the low level of fluoxetine detected in the one control water sample likely occurred due to contamination during sample processing.

#### 4.1.2 Macroinvertebrate Tissue

The mean measured exposure concentrations in macroinvertebrate tissue for the low- and high-fluoxetine treatments were 11.2 ng/g d.w. (standard deviation = 6.46, concentration range: 2.42–24.9, *n* = 9) and 130 ng/g d.w. (standard deviation = 33.3, concentration range: 12.4–398, *n* = 12), respectively (Table S4). Fluoxetine was detected in 69.2% (9/13) of samples from the low-fluoxetine treatment, and 100% of samples (12/12) from the high-fluoxetine treatment. No fluoxetine contamination was detected in the macroinvertebrate tissue samples from the control mesocosms (0/17).

### 4.2 Changes in Fish Morphology

**Figure 2:**
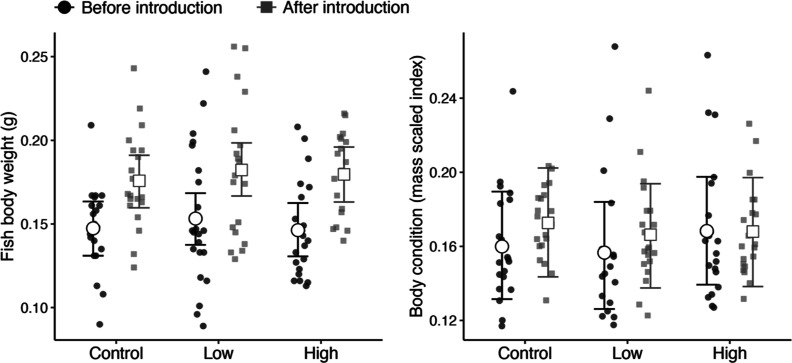
Model-predicted effects of control and fluoxetine exposure treatment on mosquitofish body weight (left) and body condition (right) before their introduction to the mesocosms (black, circles) and after their removal from the mesocosms (gray, squares). Error bars represent 95% credibility intervals. Smaller shapes represent the raw data of individual measurements used in the models.

The summary statistics for mosquitofish standard length, weight, and body condition are provided in Table S5. There were no differences among treatments in mosquitofish standard length, body weight, or body condition before being introduced into the mesocosms, or post removal from mesocosms (see Tables S6–S8 for model parameters). Irrespective of treatment, both mosquitofish length and weight increased while in the mesocosms. In the high-fluoxetine treatment, mosquitofish standard length increased by an average of 16.1% (95% CI: 7.6–23.7%), whereas mosquitofish length from the control and low-fluoxetine treatments increased by an average of 6.1% (−1.2–13.6%) and 5.6% (−1.4–12.9%), respectively. Similarly, fish weight in the high-fluoxetine treatment increased by an average of 23.1% (9.04–38.2%), and in the control and low-fluoxetine treatments it increased by 19.6% (6.33–35.6%) and 19.2% (5.7–33.1%), respectively (Figure 2, left). However, despite increases in both length and weight, we found little evidence to suggest that mosquitofish body condition improved while in the mesocosms for any of the treatments (Figure 2, right).

### 4.3 Changes in Community-Level Endpoints

#### 4.3.1 Zooplankton Abundance

The numerically dominant zooplankton taxon found across all mesocosms was copepod nauplii (Table S9), while all other taxa were in low abundance. Thus, for this analysis, we focused only on changes in total zooplankton abundance, and not diversity, among treatments and experimental stages.

**Figure 3:**
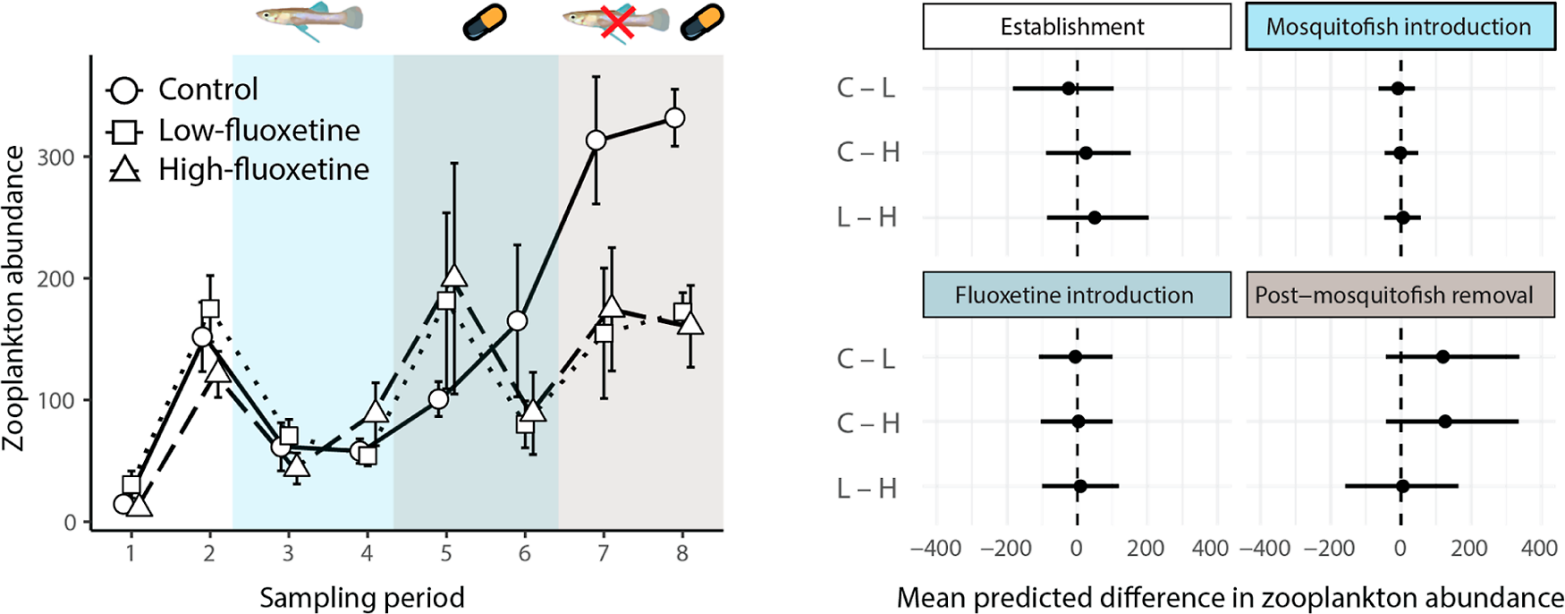
(Left side) Mean ± standard error zooplankton abundance for each sampling period of the experiment. The exposure treatments comprised a solvent control (circles, solid line), low-fluoxetine exposure (squares, dotted line) and high-fluoxetine exposure (triangles, dashed line). (Right side) Model-predicted mean difference in zooplankton abundance (with 95% credibility intervals) between the exposure treatments at each stage of the experiment (C = control, L = low fluoxetine, H = high fluoxetine).

There were no differences in zooplankton abundance among the treatment groups before the introduction of mosquitofish (i.e., at the establishment stage) or before the introduction of fluoxetine (Figure 3 and Table S10). As expected, zooplankton abundance substantially decreased in all mesocosms after the introduction of mosquitofish (Figure 2, left panel). Specifically, zooplankton abundance decreased by 61.7% (95% CI: 35.1–79.7%) in the control treatment, 61.8% (31–82.6%) in the low-fluoxetine treatment, and 53.1% (13.4–79.7%) in the high-fluoxetine treatment. However, given that we also added nutrients just before introducing mosquitofish, we cannot explicitly determine whether the observed decline in zooplankton was primarily driven by mosquitofish predation or the influx of nutrients.

From the beginning of the fluoxetine exposure period until the end of the experiment (i.e., sample periods 4–8), zooplankton abundance increased substantially more in control mesocosms than both the low- and high-fluoxetine treatments (Figure 3). Zooplankton abundance increased by 393.6% (95% CI: 46–978%) in the control mesocosms, whereas zooplankton abundance increased in low- and high-fluoxetine mesocosms by only 158.4% (−21.8 to 509%) and 173.2% (−14.4 to 528.3%), respectively. Note, however, the high uncertainty in these estimates, which is due to high variability among mesocosms and sampling weeks. Divergence in zooplankton abundance between control and fluoxetine-exposed treatments primarily occurred after the removal of mosquitofish from the mesocosms (Figure 3; right side). After the removal of mosquitofish from the mesocosms, zooplankton abundance increased by 137.9% (4.7–301.4%) in the control treatment, but only increased by 34.9% (−46.7 to 156%) in the low-fluoxetine treatment and 40.6% (−40.2 to 163.8%) in the high-fluoxetine treatment. These results suggest that fluoxetine impaired the recovery of zooplankton populations following the removal of mosquitofish.

#### 4.3.2 Biofilm Productivity

**Figure 4:**
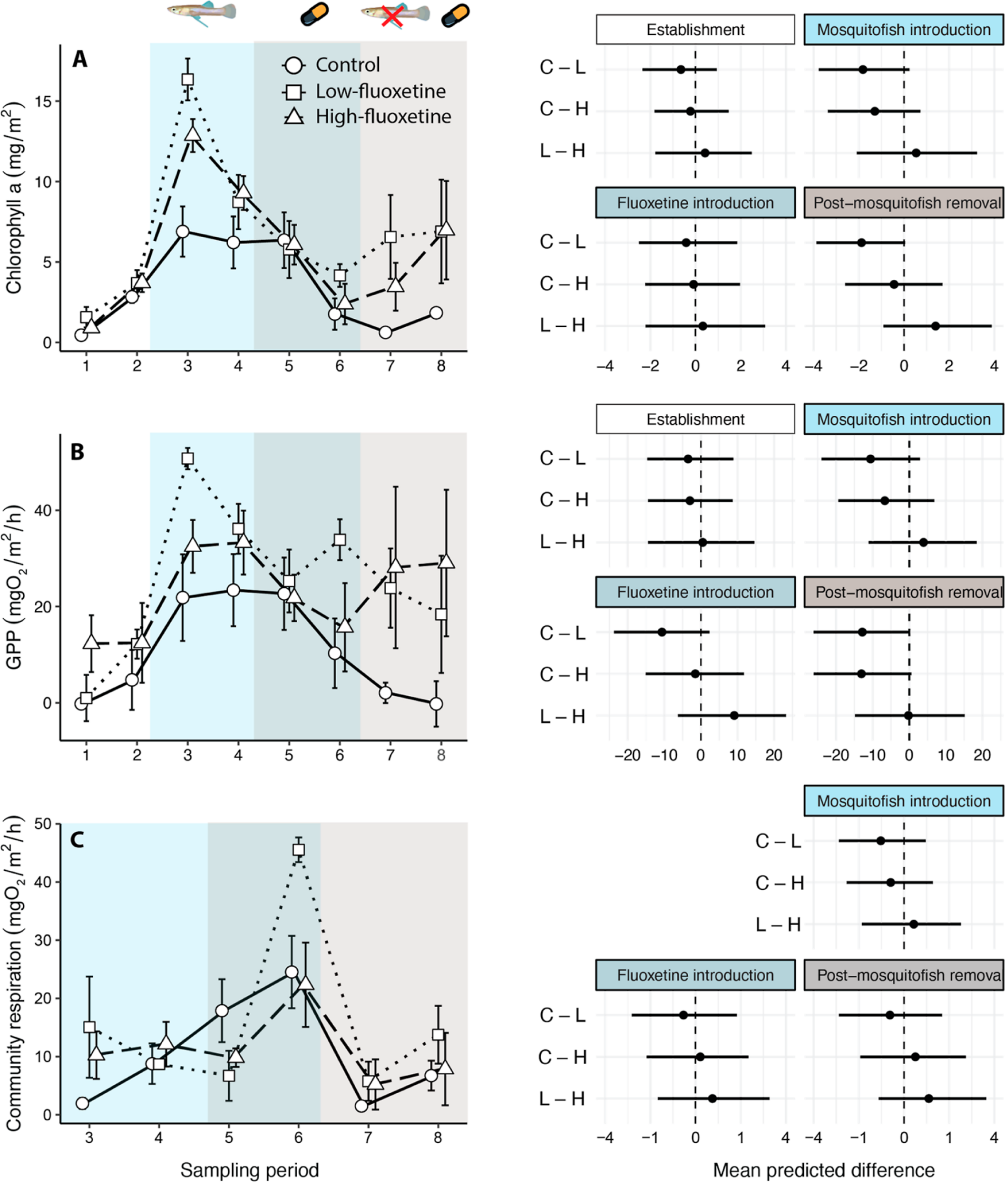
Biofilm productivity. (Left side) Mean ± standard error (A) biofilm chlorophyll *a* concentration, (B) biofilm gross primary productivity (GPP), and (C) biofilm CR for each sampling period of the experiment. Note that there was no data for biofilm CR during the establishment stage. The exposure treatments included a solvent control (circles, solid line), low-fluoxetine exposure (squares, dotted line), and high-fluoxetine exposure (triangles, dashed line). (Right side) Model-predicted mean differences (with 95% credibility intervals) among treatment groups in community response at each stage of the experiment (C = control, L = low fluoxetine, H = high fluoxetine).

The summary statistics for biofilm *Chl-a*, GPP, and CR are provided in Tables S11–S13. There was no difference in biofilm *Chl-a* or GPP among treatment groups before the introduction of mosquitofish (Tables S14–S15, Figure 4A,B). Regardless of treatment, biofilm *Chl-a* and GPP increased substantially in all mesocosms after the introduction of mosquitofish. For example, biofilm *Chl-a* increased by 70.8% (40.3–101%) in the control treatment, 84.6% (51.3–120.5%) in the low-fluoxetine treatment, and 90.4% (50.3–128%) in the high-fluoxetine treatment. Although this increase in primary productivity could partly be attributed to the introduction of mosquitofish, it is also likely that the observed spike in productivity was caused by the addition of nutrients 2 days prior to the release of mosquitofish (see Section 2.2.1). Indeed, both *Chl-a* and GPP concentrations began to decrease and converge among treatments after this initial spike (i.e., between sampling period 3 and 4, Figure 4A,B).

There were no clear differences in biofilm *Chl-a*, GPP, or CR among treatments after the introduction of fluoxetine (Figure 4). However, both *Chl-a* and GPP diverged between the control and fluoxetine-exposed treatments after the removal of mosquitofish. Compared to the control treatment, *Chl-a* concentrations were on average 47.8% (−4.49 to 100.5%) higher in the low-fluoxetine treatment, but only 7.4% (−27.4 to 42.9%) higher in the high-fluoxetine treatment. Similarly, GPP was 200.3% (2.5 to 402%) higher in the low-fluoxetine treatment and 67.5% (−2 to 136%) higher in the high-fluoxetine treatment, relative to the control treatment. Biofilm CR significantly decreased in all treatments following the removal of mosquitofish. In controls, CR decreased by 69.7% (40.9 to 98%), in the low-fluoxetine treatment by 64.4% (−31.9 to 93.7%), and in the high-fluoxetine treatment by 74.8% (44.9 to 109.4%) (Figure 4C). There was no evident treatment-by-stage interaction for biofilm CR, suggesting that there was no effect of fluoxetine on biofilm CR (Table S16).

#### 4.3.3 Seston Productivity

**Figure 5:**
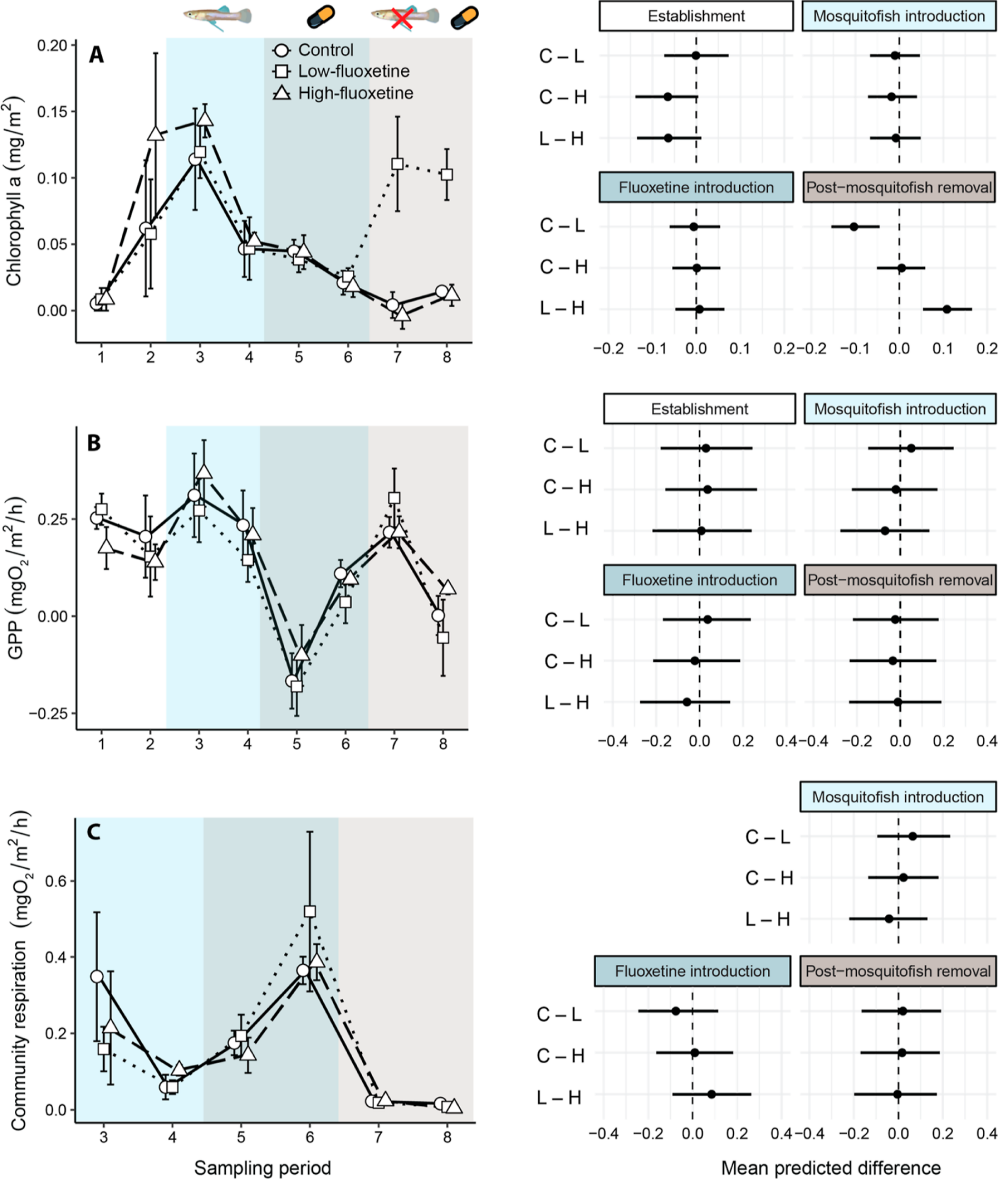
Seston productivity. (left side) Mean ± standard error (A) seston chlorophyll *a* concentration, (B) seston gross primary productivity (GPP), and (C) seston CR for each sampling period of the experiment. Note that there was no data for seston CR during the establishment stage. The exposure treatments included a solvent control (circles, solid line), low-fluoxetine exposure (squares, dotted line), and high-fluoxetine exposure (triangles, dashed line). (right side) Model-predicted mean differences (with 95% credibility intervals) among treatment groups in community response at each stage of the experiment (C = control, L = low fluoxetine, H = high fluoxetine).

The summary statistics for seston *Chl-a*, GPP, and CR are provided in Tables S17–S19. Although seston *Chl-a* was proportionally higher in the high-fluoxetine treatment in Week 2 (Figure 5), there was no statistical difference in seston *Chl-a* or GPP among the treatment groups before the introduction of mosquitofish (Tables S20 and S21). Seston *Chl-a* increased and varied substantially among all mesocosms in weeks 2 and 3 (Figure 5A), an unexpected response that was most likely caused by the addition of nutrients 2 days prior to the release of mosquitofish into the mesocosms.

There were no clear differences in seston *Chl-a*, GPP, or CR among the treatments after the introduction of fluoxetine. However, following the removal of mosquitofish from the mesocosms, seston *Chl-a* became substantially higher in the low-fluoxetine treatment relative to the other treatments (Figure 5A). Seston *Chl-a* in the low-fluoxetine treatment was 127.5% (55.1–190.8%) higher than in the control treatment, and 144.4% higher than in the high-fluoxetine treatment. Seston GPP and CR did not differ significantly among the treatment groups throughout the entire experiment (Table S21 and S22; Figure 5B,C).

#### 4.3.4 Nutrients

Summary statistics for nutrient concentrations are presented in Table S23. Overall, nutrient concentrations remained relatively stable and consistent across all treatments throughout the experiment (Tables S24 and S25). An exception occurred with a noticeable spike in ammonia (NH_3_) in the control and low-fluoxetine treatments following the introduction of the exposure (specifically on the 16th November 2020). These spikes were primarily due to substantial increases of NH_3_ in a single control mesocosm and a single low-fluoxetine mesocosm (Figure S2). Despite these outliers, there was no statistical difference in NH_3_ concentrations among treatments during this period (Table S24). Although NH_3_ levels in the two anomalous mesocosms reached potentially stressful levels for fish (∼0.1 mg L^–1^), no mosquitofish died, and no abnormalities were observed during routine checks. Ammonia concentrations returned to stable levels in all mesocosms in the subsequent sampling period (Table S23).

FRP and nitrate + nitrite (NO_*x*_) concentrations were considerably higher in the establishment stage relative to all other stages of the experiment. In fact, quantifiable concentrations of NO_*x*_ were only detected during the establishment stage. These higher levels of FRP and NO_*x*_ during the establishment stage are likely a result of the nutrient additions to stimulate productivity in the mesocosms before the introduction of mosquitofish (see Section 2.2.1).

## 5 Discussion

We found evidence that exposure to environmentally relevant levels of fluoxetine, even at low concentrations (<10 ng/L), can affect freshwater communities, and disrupt their recovery from a top-order predator. Following the introduction of mosquitofish into our experimental mesocosms, we observed a substantial decrease in zooplankton abundance, and a corresponding increase in primary productivity. However, once mosquitofish were removed from the mesocosms, planktonic abundance recovered substantially more in control mesocosms compared to mesocosms exposed to fluoxetine. By the end of the experiment, this resulted in fundamental differences in trophic structures between control and fluoxetine-treated mesocosms. Control mesocosms were characterized by higher zooplankton abundances and lower algal biomass, whereas mesocosms exposed to either low or high concentrations of fluoxetine had lower zooplankton abundances and higher algal biomass. Our study design does not allow us to quantitatively tease apart the direct effects of mosquitofish from fluoxetine on community parameters. However, given that the differences in zooplankton abundance between control and fluoxetine-treated mesocosms only emerged after the removal of mosquitofish, our results suggest that (1) fluoxetine had no apparent influence on mosquitofish consumptive effects, and (2) fluoxetine can impair the recovery of zooplankton populations after a disturbance, leading to fundamental shifts in community structure.

Our study revealed that exposure to fluoxetine, both at environmentally realistic low (∼10 ng/L) and high concentrations (∼100 ng/L), can hinder the recovery of zooplankton populations following the removal of a fish predator. In our mesocosms, zooplankton populations were dominated by copepod nauplii, the larval stage of copepods. Although mosquitofish usually target adult copepods, they will consume nauplii in the absence of larger stages.^39,40^ Fluoxetine did not appear to influence mosquitofish predation rates on nauplii, as zooplankton abundances were similar between control and fluoxetine-exposed mesocosms when mosquitofish were present. Only when mosquitofish were removed from the mesocosms—and thus also the primary predation threat—did we observe a substantial increase in zooplankton numbers in the control mesocosms relative to the fluoxetine-exposed mesocosms. Previous studies have shown that exposure to fluoxetine, and to other SSRIs, can impair the reproduction and development of planktonic species, including copepods.^31,57−59^ This suggests that fluoxetine in our study could have had effects on survival and/or other processes (e.g., reproduction and growth) that would typically allow nauplii numbers to recover following a large predation or disturbance event. However, without sufficient information on how mosquitofish and fluoxetine impacted other planktonic life stages (e.g., adult copepods), our study cannot determine the mechanisms by which fluoxetine affected zooplankton population dynamics in our mesocosms.

A recent study found that exposure to low concentrations of fluoxetine reduced copepod populations but increased the abundance of other planktonic species within experimental mesocosms.^60^ This suggests that the effects of fluoxetine may be species-dependent and/or conditional upon the sensitivity of important functional groups^23,61^ or prevailing abiotic conditions (e.g., temperature, light or oxygen availability^31,62−64^). While our study controlled for extraneous spatial and environmental factors (see section 2.1 Mesocosm Setup), we still observed high community variability—particularly in biofilm productivity—among mesocosms and within treatments even before introducing the fluoxetine exposures (e.g., see week 3; Figure 4). This initial variability in primary productivity may have influenced the observed treatment differences in zooplankton populations and community structures by the end of the experiment. These findings highlight the challenges associated with understanding the ecological effects of contaminants within complex and variable natural systems. Clearly, the effects of fluoxetine and other common pharmaceutical pollutants on zooplankton population dynamics warrants further investigation.

Following the removal of mosquitofish, the fluoxetine-exposed mesocosms had higher biofilm *Chl-a* and GPP relative to the control mesocosms. Low-fluoxetine mesocosms also had higher seston *Chl-a* than both control and high-fluoxetine mesocosms. Previous studies have shown that fluoxetine at environmentally relevant concentrations can affect algal function and photosynthesis, although the direction of observed effects has been mixed.^16,29,30,32,60^ For example,^30^ found that fluoxetine increased photosynthetic yields in two species of microalgae cultured under laboratory conditions over multiple generations. In contrast, several studies have observed negative effects of fluoxetine on biofilms, including suppressed colonisation and reduced GPP and CR.^16,29,32^ These studies, however, did not observe any changes to algal biomass, suggesting that the observed effects of fluoxetine were on biofilm metabolic functioning as opposed to algal growth. Indeed, while we observed a clear increase in biofilm and seston algal growth in fluoxetine-exposed mesocosms, this increased growth was not coupled with notable increases in CR or seston GPP, suggesting that fluoxetine may still have affected algal metabolic efficiency in our study.

Past studies investigating the effects of fluoxetine on primary producers were also conducted in the absence of fish predators. Thus, it is possible that the presence of mosquitofish in our study may have offset any potential negative effects of fluoxetine on algal productivity by regulating primary consumption. Once mosquitofish were removed from our mesocosms, the slower recovery of zooplankton in fluoxetine-exposed mesocosms likely limited grazing pressure on established phytoplankton. Indeed, density-mediated indirect effects of pollutants on ecosystems are expected to be more common than direct effects, particularly when specific functional groups or keystone species are more, or less, sensitive to contaminant effects.^65−69^ For example,^70^ found that exposure to the antibiotic norfloxacin had both direct behavioral effects and indirect predator-mediated effects on *Daphnia magna* populations, resulting in positive algal growth in a simple predator-consumer-resource food web.

Lower concentrations of fluoxetine had more pronounced effects on primary productivity, particularly seston *Chl-a*, relative to high concentrations. Nonmonotonic (or nonlinear) dose responses are increasingly reported at environmentally realistic concentrations of fluoxetine and other neuroactive pharmaceuticals, particularly for individual-level traits such as reproduction^43,71,72^ and behavior.^33,35,73,74^ On the other hand, evidence of nonmonotonic effects of pharmaceuticals on community-level endpoints are rare,^32^ but this is also likely because such studies are generally uncommon, as reviewed in.^60^ Our observed nonmonotonic responses in primary productivity could result from several different mechanisms, including receptor saturation and desensitization^75^ or environmental hormesis.^76^ Further investigations are needed to identify the potential mechanisms driving nonmonotonic responses, particularly those that allow pharmaceuticals to influence primary producers.

While changes in algal biomass are typically linked to nutrient cycles, we did not find any difference in nutrient concentrations (i.e., ammonia, FRP, NO_*x*_) between control and fluoxetine-exposed mesocosms. Nutrients were added in our mesocosms but remained generally low throughout the experiment—typical of conditions found in oligotrophic wetlands in Australia where mosquitofish are common.^39^ This may have limited our capacity to detect any significant changes in nutrients. Fluoxetine has previously been shown to adversely affect microbial communities and denitrification rates,^16,29^ suggesting that it has the potential to alter nutrient cycling. Indeed, a recent study found that ammonia and nitrates accumulated in microcosms exposed to a combination of fluoxetine and ketoprofen (an analgesic).^77^ More research is required to determine how fluoxetine and other pharmaceuticals might affect biogeochemical cycles, as we currently lack knowledge on this topic.

We observed a significant decrease in zooplankton, and a corresponding increase in primary productivity, following the introduction of mosquitofish. While we expected that mosquitofish would exert strong top–down effects, our study design does not allow us to explicitly disentangle how much these observed community changes were due to the introduction of mosquitofish and/or other environmental factors including the introduction of nutrients. Nevertheless, our results concur with prior studies that have documented high rates of predation by mosquitofish on freshwater planktonic populations, which lead to cascading effects on primary productivity.^40−42,78^ We also found that, once mosquitofish were removed from the mesocosms, planktonic populations recovered quickly, particularly in the control treatment. This suggests that mosquitofish exert important top–down effects, but that these effects can be reversed following predator removal.

We did not find any evidence that fluoxetine altered the effects of mosquitofish on community structure. Several studies have found that fluoxetine, and other SSRIs, can alter the foraging rate and activity of mosquitofish and other top-order predators in the laboratory, suggesting that these contaminants could change consumer–resource interactions in more complex natural settings,.^21,22,33,37,44,69,79,80^ Furthermore, other studies have found that the impact of predators on food-webs can be mediated by chemical contaminants (or vice versa;^22,81,82^). Yet we did not observe any effects of treatment on zooplankton abundance when fluoxetine was introduced, suggesting that there were no differences in mosquitofish foraging rates among treatments and no evidence that fluoxetine increased zooplankton vulnerability to predation. In addition, we found no evidence of an effect of fluoxetine on fish weight or body condition, suggesting that fish foraged at similar rates throughout the experiment. While the behavioral effects of fluoxetine could manifest differently in the laboratory compared to semifield and field settings,^83,84^ we did not measure mosquitofish behavior in the mesocosms, and thus cannot rule out the possibility that fluoxetine had effects on mosquitofish that were independent of their top–down pressure on zooplankton (e.g., changes in activity or schooling). It is also important to note that fluoxetine has time-dependent effects on behavior, with acute and chronic exposures potentially differing in both magnitude and direction.^85^ Therefore, prolonged exposure might lead to more pronounced disturbances in mosquitofish behavior and their consumptive effects on communities.

Our study adds to a growing body of evidence that fluoxetine, at environmentally relevant exposure concentrations, can affect freshwater organisms and their communities. Indeed, while there are limitations in extrapolating our findings to larger, more variable aquatic environments, our mesocosm study provides valuable insights into the diverse pathways by which fluoxetine can affect community-level parameters. We found no evidence that exposure to fluoxetine altered the consumptive effects of mosquitofish, but it did hinder the recovery of zooplankton populations following mosquitofish removal. This resulted in fundamental changes in the trophic dynamics of communities exposed to fluoxetine. Notably, our results suggest that fluoxetine may impact the recovery of aquatic communities to environmental disturbances. Given that the prevalence of SSRIs and other pharmaceuticals in aquatic systems is increasing around the world, this will have implications for both the ecological integrity of freshwater environments and for attempts to mitigate the impacts of other disturbances on these ecosystems (e.g., biological invasions, global warming^1^). Our study also highlights the context-dependency (e.g., species composition and sensitives; spatial and temporal environmental variation) associated with understanding the effects of pharmaceuticals in complex natural systems. As such, more community ecology studies are needed in ecotoxicology to identify general patterns and mechanisms, and to address different contaminant exposure scenarios, such as pollutant mixtures and multistressor environments.^8,19,62,86^

## Data Availability

Data and R code scripts associated with the statistical analyses are available on GitHub at following URL: https://github.com/mrmic1/FLX-invasive-community

## Supporting Information Available

All community endpoints (water quality, zooplankton, biofilm and seston productivity, nutrients), information on model structure and prior specifications, summaries of measured fluoxetine concentrations (water and macroinvertebrates), fish weight and body length summary statistics, model parameter estimates from linear mixed effect models (PDF)
