# Supplementary material for: Pharmaceutical Pollution Alters the Structure of Freshwater Communities and Hinders Their Recovery from a Fish Predator
Source: Environ Sci Technol. 2024 Jul 25;58(31):13904–17. doi: 10.1021/acs.est.4c02807 (PMC11308527; doi:10.1021/acs.est.4c02807)
Supplement: Supplementary file 1 — es4c02807_si_001.pdf [file es4c02807_si_001.pdf]

Supplementary material for: Pharmaceutical pollution alters the structure of freshwater communities and hinders their recovery from a fish predator

Marcus Michelangeli[1,2,3†], Jake M. Martin[2,3,4], Stephanie Robson[5], Daniel Cerveny[2,6], Robert Walsh[7], Erinn K. Richmond[8], Michael R. Grace[5], Jack A. Brand[2,9], Michael G. Bertram[2,3,4], Susie Ho[3], Tomas Brodin[2]*, Bob B.M. Wong[3]*

[1] School of Environment and Science, Griffith University, Nathan 4111, Australia

[2] Department of Wildlife, Fish and Environmental Studies, Swedish University of Agricultural Sciences, Umeå 901 83, Sweden

[3] School of Biological Sciences, Monash University, Melbourne 3800, Australia

[4] Department of Zoology, Stockholm University, Stockholm 114 18, Sweden

[5] Water Studies Centre, School of Chemistry, Monash University, Melbourne 3800, Australia

[6] University of South Bohemia in Ceske Budejovice, Faculty of Fisheries and Protection of Waters, South Bohemian Research Center of Aquaculture and Biodiversity of Hydrocenoses, Zatisi 728/II, Vodnany, Czech Republic

[7] Australian Waterlife, 55 Vaughan Chase, Wyndham Vale, Victoria, 3024, Australia

[8] Environmental Protection Authority Victoria, EPA Science, Macleod, Victoria, 3085, Australia

[9] Institute of Zoology, Zoological Society of London, London NW1 4RY, United Kingdom

[†] Correspondence: marcus.michelangeli@slu.se or marcus.michelangeli@monash.edu

* Co-senior authors

Summary: 30 pages, 2 figures, 25 tables

Supplementary material table of contents

Section		Pages
**1. Methods**		**S5–S6**
	**Table S1:** Mean (± standard deviation) temperature (Temp, °C), pH and dissolved oxygen (DO, mg/L) for each treatment and stage of the experiment.	S5
	**Table S2:** Model structure, number of observations (n) and prior specification for each model used in the statistical analysis.	S6
**2. Results**		
	**Table S3:** Summary statistics of detected fluoxetine concentrations (ng/L) in water samples from low- and high-fluoxetine exposure treatments. We found no water contamination in control mesocosms.	**S7**
	**Figure S1:** Mean (± SE) detected fluoxetine concentrations (ng/L) in water samples from low- and high-fluoxetine exposure treatments at each sampling date. Smaller shapes represent concentrations detected for individual mesocosms at each date.	**S8**
	**Table S4:** Summary statistics of detected fluoxetine concentrations (ng/g) in macroinvertebrate tissue samples from low- and high-fluoxetine exposure treatments. We detected no fluoxetine in macroinvertebrate tissue from control mesocosms.	**S9**
	**Table S6:** Model parameter estimates for mosquitofish standard length.	**S10**
	**Table S7:** Model parameter estimates for mosquitofish weight.	**S10**
	**Table S8:** Model parameter estimates for mosquitofish body condition.	**S10**
	**Table S9:** Summary of zooplankton taxa found in each treatment and at each stage of the experiment. Data are limited to the top 5 most abundant taxon found per experimental stage.	**S11**
	**Table S10:** Model parameter estimates for total zooplankton abundance. Values are on the log-scale.	**S12**

Table S11: Summary statistics for biofilm chlorophyll *a* concentrations (mg/m^2) for each treatment and experimental stage. **S13**

Table S12: Summary statistics for biofilm gross primary productivity ($mgO_2/m^2/h$) for each treatment and experimental stage. **S14**

Table S13: Summary statistics for biofilm community respiration ($mgO_2/m^2/h$) for each treatment and experimental stage. **S15**

Table S14: Model parameter estimates for biofilm chlorophyll *a* concentration (mg/m^2). **S16**

Table S15: Model parameter estimates for biofilm gross primary productivity ($mgO_2/m^2/h$). **S17**

Table S16: Model parameter estimates for biofilm community respiration ($mgO_2/m^2/h$). **S18**

Table S17: Summary statistics for seston chlorophyll *a* concentration (mg/L) for each treatment and experimental stage. **S19**

Table S18: Summary statistics for seston gross primary productivity ($mgO_2/L/h$) for each treatment and experimental stage. **S20**

Table S19: Summary statistics for seston community respiration ($mgO_2/L/h$) for each treatment and experimental stage. **S21**

Table S20: Model parameter estimates for seston chlorophyll *a* concentration (mg/L). **S22**

Table S21: Model parameter estimates for seston gross primary productivity ($mgO_2/L/h$). **S23**

Table S22: Model parameter estimates for seston community respiration ($mgO_2/L/h$). **S24**

Table S23: Mean (± standard deviation) concentrations of nutrients ($mg\ L^{-1}$) among treatments and experimental stages. **S25**

NH3 = ammonia, FRP = filterable reactive phosphorus, NO_x = nitric oxide, <LOQ = below limit of quantification.

Figure S2: Measured NH3 (ammonia) concentrations (mg L $^{-1}$) in water samples taken from control (blue), low-fluoxetine (yellow) and high-fluoxetine (red) mesocosm throughout the experiment. **S26**

Table S24: Model parameter estimates for ammonia concentrations (mg/L). Values are on the log-scale. **S27**

Table S25: Model parameter estimates for filterable reactive phosphorus concentrations (mg/L). Values are on the log scale. **S28**

Table S25: Model parameter estimates for filterable reactive phosphorus concentrations (mg/L). Values are on the log scale. **S29**

1. Supplementary methods

Table S1: Mean (± standard deviation) temperature (Temp, °C), pH and dissolved oxygen (DO, mg/L) for each treatment and stage of the experiment.

	Treatment	Establishment	Mosquitofish introduction	Fluoxetine introduction	Post-fish removal
**Temp**	Control	14.82 ± 3.24	17.12 ± 2.28	22.41 ± 1.23	21.46 ± 1.94
	Low	14.79 ± 3.22	17.16 ± 2.09	22.62 ± 1.58	21.80 ± 2.26
	High	14.74 ± 3.16	17.25 ± 2.09	22.97 ± 1.37	22.12 ± 2.31
**pH**	Control	7.85 ± 0.51	10.05 ± 0.97	6.71 ± 0.23	7.27 ± 0.35
	Low	7.88 ± 0.47	9.94 ± 0.93	7.04 ± 0.23	7.56 ± 0.42
	High	7.87 ± 0.50	9.87 ± 1.00	7.36 ± 0.14	7.45 ± 0.52
**DO**	Control	10.20 ± 1.84	9.90 ± 1.37	6.45 ± 0.48	6.92 ± 0.84
	Low	9.98 ± 1.67	10.06 ± 1.05	6.72 ± 0.66	7.15 ± 1.16
	High	10.09 ± 1.36	10.06 ± 1.34	6.84 ± 0.51	6.62 ± 1.21

Table S2: Model structure, number of observations (n) and prior specification for each model in our analysis.

Outcome	Structure	n	Prior
Fish standard length	Treatment * Stage + (1|Mesocosm)	120	Intercept = normal(0, 1)<br>B = normal(0,1)<br>Sigma = exponential(1)
Fish body weight	Treatment * Stage + (1|Mesocosm)	125	Intercept = normal(0, 1)<br>B = normal(0, 1)<br>Sigma = exponential(1)
Fish body condition	Treatment * Stage + (1|Mesocosm)	120	Intercept = normal(0, 1)<br>B = normal(0, 1)<br>Sigma = exponential(1)
Zooplankton abundance	Treatment * Stage + Sample date + Std average fish length + (1|Mesocosm)	84	Intercept = normal(4.5, 1)<br>B = normal(0, 1)<br>Shape = gamma(0.01, 0.01)
Chlorophyll a (biofilm)	Treatment * Stage +Sample date + Std average fish length + (1|Mesocosm)	84	Intercept = normal(5, 3)<br>B = normal(0, 1)<br>Sigma = exponential(1)
Gross primary productivity (biofilm)	Treatment * Stage + Sample date + Std average fish length + (1|Mesocosm)	84	Intercept = normal(10, 5)<br>B = normal(0, 1)<br>Sigma = exponential(1)
Community respiration (biofilm)	Treatment * Stage + Sample date + Std average fish length + (1|Mesocosm)	71	Intercept = normal(3, 1)<br>B = normal(0, 1)<br>Sigma = exponential(1)
Chlorophyll a (seston)	Treatment * Stage + Sample date + Std average fish length + (1|Mesocosm)	84	Intercept = normal(1, 0.5)<br>B = normal(0, 0.3)<br>Sigma = exponential(1)
Gross primary productivity (seston)	Treatment * Stage + Sample date + Std average fish length + (1|Mesocosm)	84	Intercept = normal(1, 0.5)<br>B = normal(0, 0.3)<br>Sigma = exponential(1)
Community respiration (seston)	Treatment * Stage + Sample date + Std average fish length + (1|Mesocosm)	72	Intercept = normal(1, 0.5)<br>B = normal(0, 0.3)<br>Sigma = exponential(1)
Aerobic respiration	Treatment * Stage + Sample date + Std average fish length + (1|Mesocosm)	144	Intercept = normal(0, 1)<br>B = normal(0, 1)<br>Sigma = exponential(1)
Ammonia (NH_3)	Treatment * Stage + Sample date + Std average fish length + (1|Mesocosm)	48	Intercept = normal(0, 1)<br>B = normal(0, 1)<br>Sigma = exponential(1)
Filterable reactive phosphorus (FRP)	Treatment * Stage + Sample date + Std average fish length + (1|Mesocosm)	48	Intercept = normal(0, 1)<br>B = normal(0, 1)<br>Sigma = exponential(1)

2. Supplementary results

Table S3: Summary statistics of detected fluoxetine concentrations (ng/L) in water samples from low- and high-fluoxetine exposure treatments. We found no water contamination in control mesocosms.

Treatment	Stage	Mean	SD	n	Min	Max
**Low**	Fluoxetine introduction	9.57	4.02	12	5.2	19
	Post-fish removal	13.05	11.38	8	5.4	39
**High**	Fluoxetine introduction	123.58	78.14	12	33.0	310
	Post-fish removal	112.00	47.46	8	43.0	200

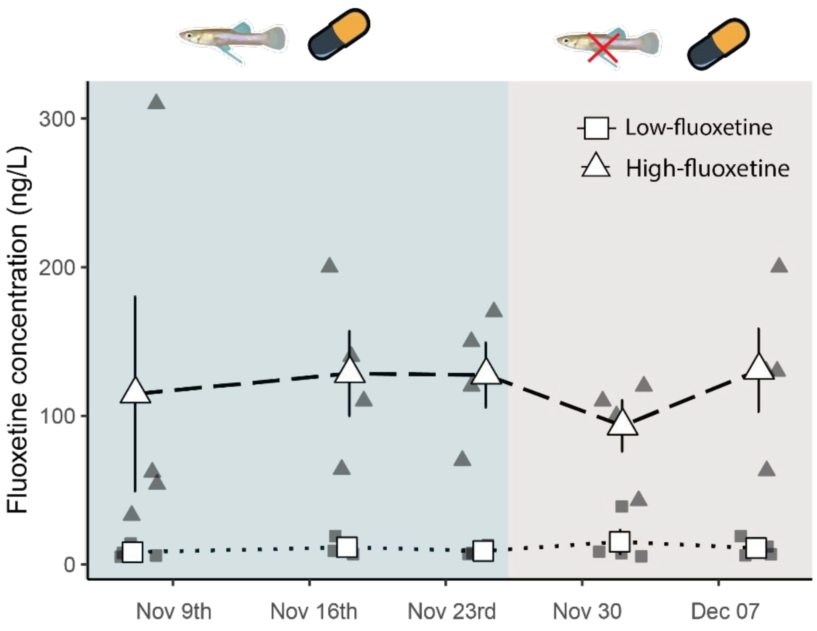

Figure S1: Mean (± SE) detected fluoxetine concentrations (ng/L) in water samples from low- and high-fluoxetine exposure treatments at each sampling date. Smaller shapes represent concentrations detected for individual mesocosms at each date.

Table S4: Summary statistics of detected fluoxetine concentrations (ng/g) in macroinvertebrate tissue samples from low- and high-fluoxetine exposure treatments. We detected no fluoxetine in macroinvertebrate tissue from control mesocosms.

Treatment	Family	Mean	SD	n	Min	Max
**Low**	Chironomidae	6.56	5.85	2	2.42	10.69
	Corixidae	14.35	7.46	4	8.77	24.85
	Notonectidae	NA	NA	0	NA	NA
	Physidae	10.02	4.77	3	6.81	15.49
**High**	Chironomidae	150.13	51.18	3	115.66	208.93
	Corixidae	220.96	250.32	2	43.96	397.97
	Notonectidae	43.86	52.07	3	12.41	103.96
	Physidae	134.10	105.11	4	58.56	289.60

Table S5: Summary statistics of mosquitofish standard length (mm), body weight (g) and body condition (mass scaled index) before introduction to the mesocosms and after removal from the mesocosms.

Size metric	Stage	Treatment	Mean	SD	n	Min	Max
**Standard length**	Before	Control	26.32	3.55	20	20.08	32.26
		Low	27.62	4.12	20	20.09	34.41
		High	25.07	3.17	20	18.06	29.57
	After	Control	27.90	2.95	20	22.16	32.89
		Low	29.14	3.33	20	22.58	34.74
		High	29.09	4.06	20	20.68	36.44
**Body weight**	Before	Control	0.15	0.03	21	0.09	0.21
		Low	0.15	0.04	22	0.09	0.24
		High	0.15	0.03	22	0.11	0.21
	After	Control	0.18	0.03	20	0.12	0.24
		Low	0.18	0.04	20	0.13	0.26
		High	0.18	0.02	20	0.14	0.22
**Body condition**	Before	Control	0.160	0.030	20	0.007	0.117
		Low	0.157	0.04	22	0.09	0.24
		High	0.168	0.038	20	0.008	0.127
	After	Control	0.173	0.020	20	0.004	0.131
		Low	0.167	0.028	20	0.006	0.123
		High	0.168	0.025	20	0.006	0.132

Table S6: Model parameter estimates for mosquitofish standard length.

Parameters	Estimate	Est.Error	l-95% CI	u-95% CI
Intercept	26.31	1.42	23.44	29.11
Treatment (Low)	1.33	2.03	-2.67	5.31
Treatment (High)	-1.25	2.00	-5.17	2.71
Stage (Post)	1.60	0.96	-0.31	3.46
Treatment (Low): Stage (Post)	-0.07	1.36	-2.69	2.69
Treatment (High): Stage (Post)	2.40	1.32	-0.16	4.99

Table S7: Model parameter estimates for mosquitofish weight.

Parameters	Estimate	Est.Error	l-95% CI	u-95% CI
Intercept	0.1474	0.0080	0.1310	0.1634
Treatment (Low)	0.0059	0.0111	-0.0157	0.0282
Treatment (High)	-0.0011	0.0113	-0.0238	0.0213
Stage (Post)	0.0284	0.0099	0.0089	0.0478
Treatment (Low): Stage (Post)	0.0006	0.0138	-0.0263	0.0285
Treatment (High): Stage (Post)	0.0050	0.0138	-0.0229	0.0322

Table S8: Model parameter estimates for mosquitofish body condition.

Parameters	Estimate	Est.Error	l-95% CI	u-95% CI
Intercept	0.1599	0.0147	0.1315	0.1895
Treatment (Low)	-0.0033	0.0208	-0.0454	0.0382
Treatment (High)	0.0083	0.0208	-0.0339	0.0485
Stage (Post)	0.0127	0.0076	-0.0021	0.0281
Treatment (Low): Stage (Post)	-0.0030	0.0106	-0.0242	0.0176
Treatment (High): Stage (Post)	-0.0130	0.0106	-0.0339	0.0078

Stage: Post = Post-mosquitofish removal from the mesocosms

Table S9: Summary of zooplankton taxa found in each treatment and at each stage of the experiment. Data are limited to the top 5 most abundant taxon found per experimental stage.

Treatment	Stage	N. taxa	Abundance	Taxon	N
**Control**	Establishment	4	84	Copepoda	77
				Cyclopoida	4
				Calanoida	2
				Ostracoda	2
				Cladocera	1
	Fish introduction	3	60	Copepoda	57
				Calanoida	2
				Cyclopoida	2
				Ostracoda	1
				Collembolla	1
	Fluoxetine introduction	5	133	Copepoda	123
				Cyclopoida	7
				Cladocera	2
				Ostracoda	2
				Hyrdidae	1
	Post-mosquitofish removal	5	323	Copepoda	308
				Cyclopoida	8
				Ostracoda	4
				Cladocera	3
				Calanoida	1
**Low**	Establishment	5	103	Copepoda	93
				Cyclopoida	5
				Calanoida	4
				Ostracoda	2
				Arachinida	1
				Copepoda	59
	Fish introduction	4	63	Calanoida	2
				Cyclopoida	2
				Oligochaeta	1
				Ostracoda	1
	Fluoxetine introduction	4	131	Copepoda	124
				Cyclopoida	5
				Cladocera	2
				Calanoida	1
				Ostracoda	1
	Post-mosquitofish removal	5	164	Copepoda	152
				Cyclopoida	6
				Cladocera	3
				Calanoida	2
				Ostracoda	1

Treatment	Stage	N. taxa	Abundance	Taxon	N
**High**	Establishment	3	d66	Copepoda	60
				Cyclopoida	3
				Calanoida	2
				Ostracoda	2
				Collembolla	1
	Fish introduction	4	66	Copepoda	62
				Calanoida	2
				Cyclopoida	2
				Diptera	2
				Ostracoda	1
	Fluoxetine introduction	4	145	Copepoda	130
				Cyclopoida	9
				Cladocera	4
				Nematode	1
				Oligochaeta	1
	Post-mosquitofish removal	6	168	Copepoda	147
				Cyclopoida	14
				Calanoida	5
				Cladocera	2
				Ostracoda	1

Table S10: Model parameter estimates for total zooplankton abundance. Values are on the log-scale.

Parameters	Estimate	Est.Error	l-95% CI	u-95% CI
Intercept	5.05	0.37	4.35	5.79
Stage (Fish intro)	-0.96	0.30	-1.53	-0.39
Stage (Flx intro)	-0.23	0.41	-1.03	0.61
Stage (Fish removal)	0.63	0.56	-0.48	1.75
Treatment (Low)	0.13	0.37	-0.60	0.87
Treatment (High)	-0.18	0.38	-0.92	0.58
Sample week	0.00	0.10	-0.20	0.20
Average fish length	-0.16	0.13	-0.42	0.10
Stage (Fish intro): Treatment (Low)	0.00	0.39	-0.76	0.75
Stage (Flx intro): Treatment (Low)	-0.09	0.40	-0.87	0.71
Stage (Fish removal): Treatment (Low)	-0.65	0.39	-1.40	-0.13
Stage (Fish intro): Treatment (High)	0.21	0.40	-0.57	0.98
Stage (Flx intro): Treatment (High)	0.15	0.39	-0.64	0.93
Stage (Fish removal): Treatment (High)	-0.37	0.39	-1.14	0.39

Stages: Fish intro = Mosquitofish introduction; Flx intro = Fluoxetine introduction; Fish removal = post-mosquitofish removal.

Table S11: Summary statistics for biofilm chlorophyll *a* concentrations (mg/m^2) for each treatment and experimental stage.

Treatment	Stage	Mean	SD	n	Min	Max
**Control**	Establishment	2.83	0.75	4	2.13	3.84
	Fish introduction	6.55	2.96	8	1.70	9.45
	Fluoxetine introduction	4.06	3.58	8	0.11	9.46
	Post-mosquitofish removal	1.23	0.85	8	0.26	2.47
**Low**	Establishment	3.68	1.61	4	2.32	5.64
	Fish introduction	12.54	4.94	8	6.42	19.53
	Fluoxetine introduction	4.97	2.64	8	1.10	9.24
	Post-mosquitofish removal	6.73	5.43	8	1.39	15.90
**High**	Establishment	3.70	1.15	4	2.46	5.24
	Fish introduction	11.07	2.71	8	7.35	15.16
	Fluoxetine introduction	4.23	3.04	8	0.88	9.59
	Post-mosquitofish removal	5.22	4.83	8	0.19	13.32

Table S12: Summary statistics for biofilm gross primary productivity ($mgO_2/m^2/h$) for each treatment and experimental stage.

Treatment	Stage	Mean	SD	n	Min	Max
**Control**	Establishment	4.76	12.44	4	-7.24	20.81
	Fish introduction	22.64	15.36	8	-1.18	40.15
	Fluoxetine introduction	16.49	15.17	8	-3.05	36.47
	Post-mosquitofish removal	0.92	6.88	8	-13.12	8.84
**Low**	Establishment	12.21	6.05	4	4.11	17.06
	Fish introduction	43.46	10.74	8	23.77	57.15
	Fluoxetine introduction	29.60	11.19	8	8.85	44.37
	Post-mosquitofish removal	21.11	19.46	8	-6.38	51.85
**High**	Establishment	12.46	16.57	4	-7.49	26.10
	Fish introduction	32.90	11.28	8	19.33	52.98
	Fluoxetine introduction	18.75	13.95	8	1.29	42.54
	Post-mosquitofish removal	28.58	29.68	8	-2.48	66.87

Table S13: Summary statistics for biofilm community respiration ($mgO_2/m^2/h$) for each treatment and experimental stage.

Treatment	Stage	Mean	SD	n	Min	Max
**Control**	Fish introduction	5.37	5.95	8	0.43	17.03
	Fluoxetine introduction	21.21	11.35	8	2.98	43.12
	Post-mosquitofish removal	4.12	4.45	8	0.04	13.70
**Low**	Fish introduction	11.88	11.92	8	1.22	40.43
	Fluoxetine introduction	23.35	21.73	7	0.37	47.87
	Post-mosquitofish removal	9.76	8.94	8	0.10	28.50
**High**	Fish introduction	11.19	7.39	8	1.99	23.32
	Fluoxetine introduction	16.08	11.79	8	5.28	40.33
	Post-mosquitofish removal	6.53	10.01	8	0.22	26.32

Table S14: Model parameter estimates for biofilm chlorophyll *a* concentration (mg/m^2).

**Parameters**	**Estimate**	**Est.Error**	**l-95% CI**	**u-95% CI**
Intercept	6.82	1.94	3.05	10.64
Stage (Fish intro)	7.14	1.63	3.94	10.29
Stage (Flx intro)	7.03	2.39	2.38	11.71
Stage (Fish removal)	7.92	3.31	1.51	14.36
Treatment (Low)	-0.20	2.35	-4.62	4.56
Treatment (High)	0.23	2.28	-4.36	4.74
Sample week	-1.74	0.55	-2.81	-0.67
Average fish length	0.90	0.74	-0.49	2.40
Stage (Fish intro): Treatment (Low)	3.54	1.99	-0.27	7.58
Stage (Flx intro): Treatment (Low)	1.14	2.03	-2.80	5.12
Stage (Fish removal): Treatment (Low)	5.93	2.02	1.98	9.79
Stage (Fish intro): Treatment (High)	2.14	1.99	-1.79	5.91
Stage (Flx intro): Treatment (High)	0.18	2.04	-3.79	4.20
Stage (Fish removal): Treatment (High)	2.74	1.98	-1.10	6.62

Stages: Fish intro = Mosquitofish introduction; Flx intro = Fluoxetine introduction; Fish removal = post-mosquitofish removal.

Table S15: Model parameter estimates for biofilm gross primary productivity ($mgO_2/m^2/h$).

**Parameters**	**Estimate**	**Est.Error**	**l-95% CI**	**u-95% CI**
Intercept	10.23	5.64	-1.14	21.16
Stage (Fish intro)	16.11	3.91	8.61	23.86
Stage (Flx intro)	11.27	4.90	1.42	20.72
Stage (Fish removal)	-0.10	6.70	-13.48	12.87
Treatment (Low)	3.49	5.99	-8.53	15.12
Treatment (High)	3.00	5.97	-8.90	14.27
Sample week	-0.78	1.29	-3.29	1.73
Average fish length	5.00	2.83	-0.71	10.62
Stage (Fish intro): Treatment (Low)	7.09	5.19	-3.02	17.01
Stage (Flx intro): Treatment (Low)	7.14	5.02	-2.93	16.78
Stage (Fish removal): Treatment (Low)	9.33	5.06	0.82	19.06
Stage (Fish intro): Treatment (High)	3.69	5.16	-6.42	13.91
Stage (Flx intro): Treatment (High)	-1.48	5.09	-11.40	8.55
Stage (Fish removal): Treatment (High)	10.06	5.15	0.05	20.04

Stages: Fish intro = Mosquitofish introduction; Flx intro = Fluoxetine introduction; Fish removal = post-mosquitofish removal.

Table S16: Model parameter estimates for biofilm community respiration ($mgO_2/m^2/h$).

**Parameters**	**Estimate**	**Est.Error**	**l-95% CI**	**u-95% CI**
Intercept	0.64	0.83	-0.96	2.31
Stage (Flx intro)	0.83	0.50	-0.13	1.85
Stage (Fish removal)	-2.07	0.74	-3.46	-0.55
Treatment (Low)	0.52	0.49	-0.43	1.53
Treatment (High)	0.29	0.48	-0.69	1.25
Sample week	0.49	0.19	0.10	0.87
Average fish length	0.37	0.20	-0.02	0.76
Stage (Flx intro): Treatment (Low)	-0.24	0.61	-1.42	0.97
Stage (Fish removal): Treatment (Low)	-0.20	0.59	-1.38	0.95
Stage (Flx intro): Treatment (High)	-0.40	0.57	-1.51	0.77
Stage (Fish removal): Treatment (High)	-0.55	0.60	-1.74	0.62

Stages: Flx intro = Fluoxetine introduction; Fish removal = post-mosquitofish removal.

Table S17: Summary statistics for seston chlorophyll *a* concentrations (mg/L) for each treatment and experimental stage.

Treatment	Stage	Mean	SD	n	Min	Max
**Control**	Establishment	0.06	0.10	4	0.00	0.21
	Fish introduction	0.08	0.07	8	0.00	0.21
	Fluoxetine introduction	0.03	0.02	8	0.00	0.06
	Post-mosquitofish removal	0.01	0.01	8	-0.02	0.02
**Low**	Establishment	0.06	0.08	4	0.01	0.18
	Fish introduction	0.08	0.06	8	0.00	0.17
	Fluoxetine introduction	0.03	0.02	8	0.01	0.06
	Post-mosquitofish removal	0.11	0.05	8	0.01	0.18
**High**	Establishment	0.13	0.12	4	0.02	0.26
	Fish introduction	0.10	0.05	8	0.03	0.17
	Fluoxetine introduction	0.03	0.02	8	0.00	0.07
	Post-mosquitofish removal	0.00	0.02	8	-0.02	0.03

Table S18: Summary statistics for seston gross primary productivity ($mgO_2/L/h$) for each treatment and experimental stage.

Treatment	Stage	Mean	SD	n	Min	Max
**Control**	Establishment	0.21	0.21	4	-0.09	0.39
	Fish introduction	0.27	0.19	8	-0.01	0.58
	Fluoxetine introduction	-0.03	0.18	8	-0.32	0.19
	Post-mosquitofish removal	0.11	0.14	8	-0.09	0.33
**Low**	Establishment	0.15	0.21	4	0.03	0.46
	Fish introduction	0.21	0.15	8	0.02	0.43
	Fluoxetine introduction	-0.07	0.17	8	-0.37	0.13
	Post-mosquitofish removal	0.12	0.25	8	-0.28	0.44
**High**	Establishment	0.14	0.09	4	0.03	0.24
	Fish introduction	0.29	0.17	8	0.01	0.53
	Fluoxetine introduction	0.00	0.15	8	-0.24	0.13
	Post-mosquitofish removal	0.14	0.10	8	0.03	0.31

Table S19: Summary statistics for seston community respiration ($mgO_2/L/h$) for each treatment and experimental stage.

Treatment	Stage	Mean	SD	n	Min	Max
**Control**	Fish introduction	0.20	0.27	8	0.00	0.74
	Fluoxetine introduction	0.11	0.10	8	0.01	0.27
	Post-mosquitofish removal	0.16	0.20	8	0.03	0.66
**Low**	Fish introduction	0.27	0.12	8	0.10	0.42
	Fluoxetine introduction	0.36	0.33	8	0.07	1.15
	Post-mosquitofish removal	0.26	0.16	8	0.06	0.52
**High**	Fish introduction	0.02	0.01	8	0.00	0.03
	Fluoxetine introduction	0.01	0.01	8	0.00	0.03
	Post-mosquitofish removal	0.01	0.02	8	0.00	0.04

Table S20: Model parameter estimates for seston chlorophyll *a* concentration (mg/L).

Parameters	Estimate	Est.Error	l-95% CI	u-95% CI
Intercept	0.12	0.04	0.05	0.19
Stage (Fish intro)	0.06	0.03	-0.01	0.12
Stage (Flx intro)	0.07	0.05	-0.03	0.17
Stage (Fish removal)	0.10	0.07	-0.03	0.24
Treatment (Low)	0.00	0.04	-0.07	0.07
Treatment (High)	0.07	0.04	-0.01	0.14
Sample week	-0.03	0.01	-0.05	-0.01
Average fish length	-0.01	0.01	-0.03	0.00
Stage (Fish intro): Treatment (Low)	0.01	0.04	-0.08	0.09
Stage (Flx intro): Treatment (Low)	0.00	0.04	-0.08	0.09
Stage (Fish removal): Treatment (Low)	0.10	0.04	0.02	0.19
Stage (Fish intro): Treatment (High)	-0.05	0.04	-0.13	0.04
Stage (Flx intro): Treatment (High)	-0.07	0.04	-0.15	0.02
Stage (Fish removal): Treatment (High)	-0.07	0.04	-0.15	0.01

Stages: Fish intro = Mosquitofish introduction; Flx intro = Fluoxetine introduction; Fish removal = post-mosquitofish removal.

Table S21: Model parameter estimates for seston gross primary productivity ($mgO_2/L/h$).

Parameters	Estimate	Est.Error	l-95% CI	u-95% CI
Intercept	0.26	0.11	0.06	0.48
Stage (Fish intro)	0.13	0.08	-0.03	0.29
Stage (Flx intro)	-0.10	0.12	-0.33	0.13
Stage (Fish removal)	0.11	0.16	-0.21	0.42
Treatment (Low)	-0.03	0.11	-0.24	0.18
Treatment (High)	-0.04	0.11	-0.25	0.17
Sample week	-0.04	0.03	-0.09	0.02
Average fish length	-0.01	0.04	-0.08	0.06
Stage (Fish intro): Treatment (Low)	-0.02	0.11	-0.23	0.20
Stage (Flx intro): Treatment (Low)	-0.01	0.11	-0.23	0.21
Stage (Fish removal): Treatment (Low)	0.05	0.11	-0.16	0.27
Stage (Fish intro): Treatment (High)	0.06	0.11	-0.16	0.27
Stage (Flx intro): Treatment (High)	0.06	0.11	-0.15	0.27
Stage (Fish removal): Treatment (High)	0.07	0.11	-0.15	0.29

Stages: Fish intro = Mosquitofish introduction; Flx intro = Fluoxetine introduction; Fish removal = post-mosquitofish removal.

Table S22: Model parameter estimates for seston community respiration ($mgO_2/L/h$).

Parameters	Estimate	Est.Error	l-95% CI	u-95% CI
Intercept	0.14	0.14	-0.15	0.42
Stage (Flx intro)	0.06	0.10	-0.14	0.27
Stage (Fish removal)	-0.20	0.15	-0.51	0.10
Treatment (Low)	-0.06	0.08	-0.23	0.10
Treatment (High)	-0.02	0.08	-0.18	0.14
Sample week	0.01	0.04	-0.06	0.08
Average fish length	0.00	0.02	-0.05	0.05
Stage (Flx intro): Treatment (Low)	0.14	0.11	-0.08	0.35
Stage (Fish removal): Treatment (Low)	0.04	0.11	-0.18	0.26
Stage (Flx intro): Treatment (High)	0.01	0.11	-0.20	0.23
Stage (Fish removal): Treatment (High)	0.01	0.11	-0.20	0.22

Stages: Flx intro = Fluoxetine introduction; Fish removal = post-mosquitofish removal.

Table S23: Mean (± standard deviation) concentrations of nutrients (mg L $^{-1}$) among treatments and experimental stages. NH3 = ammonia, FRP = filterable reactive phosphorus, NO_x = nitric oxide, <LOQ = below limit of quantification.

	Treatment	Establishment	Mosquitofish introduction	Fluoxetine introduction	Post-fish removal
**NH3**	Control	0.0050 ± 0.0014	0.0063 ± 0.0015	0.0448 ± 0.0530	0.0045 ± 0.0006
	Low	0.0068 ± 0.0026	0.0060 ± 0.0018	0.0405 ± 0.0411	0.0037 ± 0.0015
	High	0.0045 ± 0.0013	0.0060 ± 0.0014	0.0104 ± 0.0104	0.0037 ± 0.0013
**FRP**	Control	0.0305 ± 0.0095	0.0025 ± 0.0006	0.0020 ± 0.0000	0.0023 ± 0.0005
	Low	0.0330 ± 0.0048	0.0020 ± 0.0008	0.0018 ± 0.0005	0.0020 ± 0.0000
	High	0.0257 ± 0.0161	0.0023 ± 0.0005	0.0017 ± 0.0006	0.0020 ± 0.0000
**NO_x**	Control	1.0500 ± 0.0577	<LOQ	<LOQ	<LOQ
	Low	1.1250 ± 0.0500	<LOQ	<LOQ	<LOQ
	High	1.0450 ± 0.0640	<LOQ	<LOQ	<LOQ

Figure S2: Measured NH3 (ammonia) concentrations (mg L $^{-1}$) in water samples taken from control (blue), low-fluoxetine (yellow) and high-fluoxetine (red) mesocosm throughout the experiment.

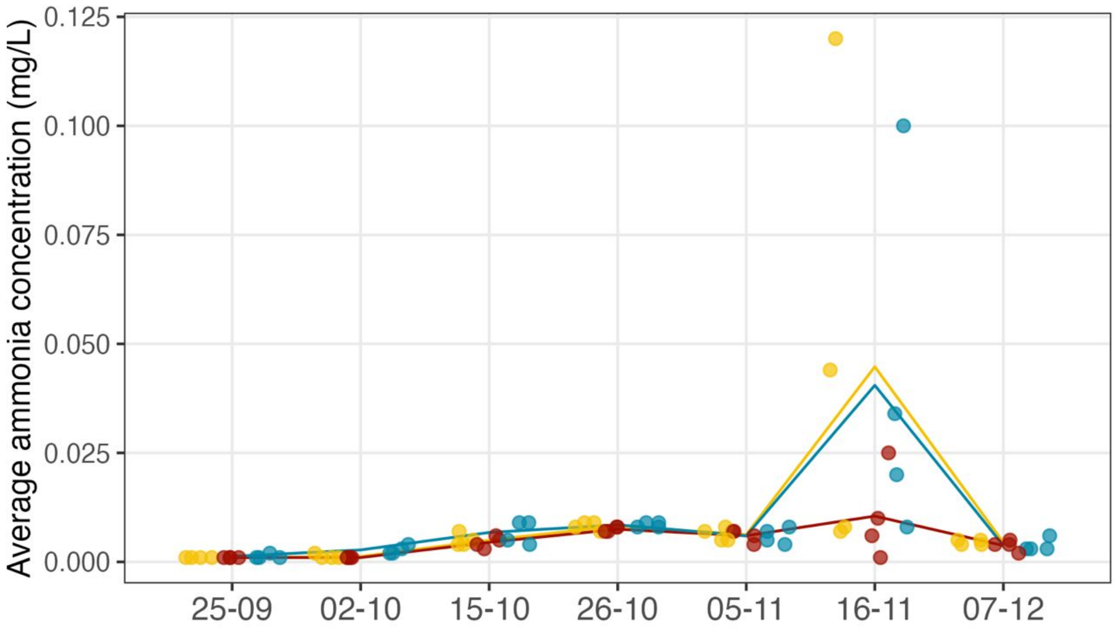

Table S24: Model parameter estimates for ammonia concentrations (mg/L). Values are on the log-scale.

Parameters	Estimate	Est.Error	l-95% CI	u-95% CI
Intercept	-2.48	0.62	-3.71	-1.28
Stage (Fish intro)	-0.05	0.41	-0.85	0.73
Stage (Flx intro)	0.44	0.57	-0.66	1.56
Stage (Fish removal)	-0.32	0.74	-1.80	1.13
Treatment (Low)	0.07	0.22	-0.36	0.50
Treatment (High)	-0.08	0.22	-0.52	0.33
Sample week	0.07	0.19	-0.31	0.45
Average fish length	0.04	0.06	-0.09	0.16
Stage (Fish intro): Treatment (Low)	-0.11	0.29	-0.69	0.47
Stage (Flx intro): Treatment (Low)	0.00	0.29	-0.55	0.56
Stage (Fish removal): Treatment (Low)	-0.18	0.29	-0.75	0.41
Stage (Fish intro): Treatment (High)	0.05	0.29	-0.49	0.64
Stage (Flx intro): Treatment (High)	-0.50	0.28	-1.05	0.07
Stage (Fish removal): Treatment (High)	-0.02	0.29	-0.57	0.57

Stages: Fish intro = Mosquitofish introduction; Flx intro = Fluoxetine introduction; Fish removal = post-mosquitofish removal.

Table S25: Model parameter estimates for filterable reactive phosphorus concentrations (mg/L). Values are on the log scale.

**Parameters**	**Estimate**	**Est.Error**	**l-95% CI**	**u-95% CI**
Intercept	-0.50	0.59	-1.65	0.66
Stage (Fish intro)	-0.40	0.39	-1.15	0.34
Stage (Flx intro)	-0.15	0.58	-1.27	0.97
Stage (Fish removal)	0.23	0.77	-1.26	1.73
Treatment (Low)	0.02	0.11	-0.20	0.24
Treatment (High)	-0.13	0.11	-0.35	0.09
Sample week	-0.34	0.19	-0.71	0.03
Average fish length	0.04	0.03	-0.02	0.11
Stage (Fish intro): Treatment (Low)	-0.16	0.14	-0.43	0.10
Stage (Flx intro): Treatment (Low)	-0.12	0.14	-0.38	0.15
Stage (Fish removal): Treatment (Low)	-0.09	0.14	-0.36	0.18
Stage (Fish intro): Treatment (High)	0.09	0.14	-0.19	0.35
Stage (Flx intro): Treatment (High)	0.02	0.13	-0.23	0.29
Stage (Fish removal): Treatment (High)	0.09	0.14	-0.18	0.35

Stages: Fish intro = Mosquitofish introduction; Flx intro = Fluoxetine introduction; Fish removal = post-mosquitofish removal.
